# Heavy metals phyto-assessment in commonly grown vegetables: water spinach (*I. aquatica*) and okra (*A. esculentus*)

**DOI:** 10.1186/s40064-016-2125-5

**Published:** 2016-04-16

**Authors:** Chuck Chuan Ng, Md Motior Rahman, Amru Nasrulhaq Boyce, Mhd Radzi Abas

**Affiliations:** Faculty of Science, Institute of Biological Sciences, University of Malaya, Kuala Lumpur, Malaysia; Department of Plant Agriculture, Ontario Agricultural College, University of Guelph, Ontario, Canada; Chemistry Department, Faculty of Science, University of Malaya, Kuala Lumpur, Malaysia

**Keywords:** Water spinach, Okra, Heavy metal accumulation, Contamination

## Abstract

The growth response, metal tolerance and phytoaccumulation properties of water spinach (*Ipomoea aquatica*) and okra (*Abelmoschus esculentus*) were assessed under different contaminated spiked metals: control, 50 mg Pb/kg soil, 50 mg Zn/kg soil and 50 mg Cu/kg soil. The availability of Pb, Zn and Cu metals in both soil and plants were detected using flame atomic absorption spectrometry. The concentration and accumulation of heavy metals from soil to roots and shoots (edible parts) were evaluated in terms of translocation factor, accumulation factor and tolerance index. Okra recorded the highest accumulation of Pb (80.20 mg/kg) in its root followed by Zn in roots (35.70 mg/kg) and shoots (34.80 mg/kg) of water spinach, respectively. Different accumulation trends were observed with, Pb > Zn > Cu in okra and Zn > Pb > Cu in water spinach. Significant differences (*p* < 0.01) of Pb, Zn and Cu accumulation were found in both water spinach and okra cultivated among tested treatments. However, only the accumulation of Pb metal in the shoots of water spinach and okra exceeded the maximum permissible levels of the national Malaysian Food Act 1983 and Food Regulations 1985 (2006) as well as the international Codex Alimentarius Commission limits. This study has shown that both water spinach and okra have good potential as Pb and Zn phytoremediators.

## Background

Water spinach and okra are readily available tropical vegetables found in many countries located across the equator region. Both water spinach and okra shared similar ecological vegetable properties as they are commonly grown edible plants. Both vegetables contain high amounts of vitamins and minerals such as phosphorus, magnesium, calcium, potassium and others which are required in our diet for a healthy living (Singh et al. 2010) and are often regarded as the daily staple diet for many people. Vegetables able to provide energy as it consists most of the essential nutrients, such as proteins, carbohydrates, minerals, vitamins and other trace elements (Itanna 2002). Even though vegetables are an important component of our daily diet, there is little information available as to its contamination by heavy metals. A common example of contamination includes bioaccumulation of heavy metals in vegetables. Bioaccumulation refers to the increase in concentration of a particular chemical or element in biological organisms over time and can pose a threat to the well-being of plants, animals and human beings (Sharma et al. 2006; Shi and Cai 2009). It is well documented that heavy metals inhibit many enzymes and thus able to disrupt metabolic processes, including photosynthesis in plants.

Most people assume that all vegetables are nutritious as well as safe to consume, unaware that some parts of the vegetable may be contaminated with heavy metals and other sources of contaminants. Heavy metals are non-biodegradable and can be very persistent in the environment; have the potential to accumulate in different body organs (Radwan and Salama 2006; Chailapakul et al. 2008; Qishlaqi et al. 2008). By consuming contaminated vegetables, excessive accumulation of dietary heavy metals such as cadmium, lead and chromium can lead to severe health problems in humans (Calderon et al. 2003; Li et al. 2009; Woimant and Trocello 2014). Heavy metals contamination in food specifically in vegetables, have been reported in many countries including Malaysia and other Asia–Pacific countries (Nadal et al. 2005; Nordin and Selamat 2013). Mohamed et al. (2003) and Sharma et al. (2007) revealed that heavy metals have different effects on various vegetable plants. Plants have the ability to accumulate metals from the environment and can be categorized as unsafe for consumption if the plants are cultivated on or near to contaminated land. Plants require many sorts of essential macro and micro (trace) mineral nutrients for normal growth and development and these include nitrogen, phosphorus, potassium, magnesium, calcium, iron, zinc and sulphur. However, vegetable plants such as water spinach and okra can easily absorb and take in heavy metals naturally into their vacuoles (Ismail et al. 2005). It has been reported that both lead and cadmium are the most common heavy metals in soils while all other types of heavy metals are significantly toxic in high concentration amounts (Radwan and Salama 2006). The ingestion of vegetables grown in such contaminated soils will pose a danger to both animal and human health.

The aims of this study were to (1) determine the responses of heavy metals toxic effect; and to (2) evaluate the environmental and health aspects of heavy metals accumulation for both water spinach and okra grown under contaminated soil conditions. The growth performance, metal tolerance and metal accumulation properties of both water spinach and okra were assessed while the obtained metals accumulation were evaluated in light of the permissible levels of metal concentrations stipulated by the Malaysian Food Act 1983 and Food Regulations 1985 (2006) together with the Codex Alimentarius Commission–Joint FAO/WHO Expert Committee on Food Additives (JECFA) standards.

## Methods

### Samplings and experimental design

The study was conducted in the glasshouse of the Institute of Biological Sciences, Faculty of Science, University of Malaya, Kuala Lumpur, Malaysia using pot assays with the average room temperature in the region of 31 °C throughout the day. Top soil (0–20 cm) was collected at 3°79′N latitude and 101°12′E longitude, then sieved at <4 mm and thoroughly mixed to produce a homogenous soil composite. All of the soil was air-dried for a week before being artificially spiked with 50 mg/kg metal salts of Pb, Zn and Cu using Pb(NO_3_)_2_, ZnSO_4_ and CuSO_4_, respectively. Each pot of 0.1 m × 0.12 m area size was then filled up with two kilograms of soil with different treatments of spiked metals: control, Pb (50 mg Pb/kg soil), Zn (50 mg Zn/kg soil) and Cu (50 mg Cu/kg soil). The concentrations of artificially spiked metal treatments were prepared based on the Malaysian guidelines for soil contamination (DOE 2009) and the European Union heavy metals threshold limits (Lado et al. 2008) which exceeding the median permissible natural occurring levels. The preliminary soil assessment and initial concentrations of Pb, Zn and Cu metals (Table 1) from the collected control and spiked soils were examined using flame atomic absorption spectrometry (FAAS). The soil texture of the growth media composed of 71.6 % clay, 3.9 % silt and 24.5 % sand. The texture of the soil is an essential aspect for plant growth as it influences the soil fertility, soil porosity, soil stability, ease of tillage and nutrient retention. Both water spinach and okra seeds provided by the Malaysian Agricultural Research and Development Institute (MARDI) were sown in control soil for about 14 days to acclimatize the seedlings. The seedlings were then transferred into plastic pots and each pot was evenly watered with 50 ml of glasshouse tap water once a day. Soil pH and growth parameters such as number of leaves and plant height were measured throughout the experiments. Water spinach and okra were tested as individual experiments. Treatments of both experiments were conducted under the completely randomized design (CRD) with three replications.

**Table 1:** Preliminary growth media soil parameters

Characteristics (units)	Mean ± SD
Soil texture	
Sand (%)	24.5
Silt (%)	3.9
Clay (%)	71.6
Soil pH	5.06 ± 0.43
Soil moisture content (%)	19.41 ± 3.62
Soil metal contents (mg/kg)	
Pb	1.23 ± 0.19
Zn	0.41 ± 0.05
Cu	0.55 ± 0.01

*SD* standard deviation

### Soil and plant analysis

The procedure of plant analysis was modified from Atayese et al. (2009) according to the Method 7000B (US EPA 2007) where freshly harvested plants were brought into the laboratory and washed in running water followed by distilled water to remove soil particles and any air-borne pollutants. The plants were divided into shoots (stems and leaves) and roots and weighed to determine the fresh weight before being cut into small pieces with a plastic knife. The plant samples were subsequently oven-dried at 75 °C for 48 h until it achieved a constant weight and weighed once again and then homogenized using a mortar and pestle. Approximately, 0.5 g of homogenized powder (shoot or root) was transferred into a 100 ml conical flask and 5 ml of concentrated H_2_SO_4_ added, followed by 25 ml of concentrated HNO_3_ and 5 ml of concentrated HCl in a conical flask. The contents of the conical flask was then heated at 200 °C for 1 h in a fume cupboard and cooled down to room temperature. After cooling, 20 ml of distilled water was added and the mixture was filtered using filter paper number one (110 mm). Subsequently, the mixture was transferred into a 50 ml volumetric flask and distilled water added up till to the mark. Finally, the volumetric flask was left to settle down for 15 h. The supernatant obtained was then analyzed for total Pb, Zn and Cu metal concentrations using the Perkin-Elmer AAnalyst 400 flame atomic absorption spectrometry (FAAS). Soil samples were air-dried for 48 h until it reached a constant weight before being subjected to similar analysis procedures. The precision of the chemical analysis technique was controlled using the BAM Germany (BRM#12-mixed sandy soil) certified reference material and the results are reported in Table 2.

**Table 2:** Certified reference material (CRM) and metal recovery (%) for Pb, Zn and Cu metals

Heavy metals	Initial soil (mg/kg)	Spiked metal (mg/kg)	CRM (mg/kg)^a^	Measured (mg/kg)	Metal recovery (%)
Pb	1.23 ± 0.19	50.24 ± 0.38	204.0 ± 6.0	219.44 ± 17.06	107.57
Zn	0.41 ± 0.05	50.83 ± 0.86	380.0 ± 13.0	356.06 ± 19.26	93.70
Cu	0.55 ± 0.01	49.62 ± 0.22	80.7 ± 3.5	76.73 ± 10.61	95.08

Mean ± standard deviation

^a^BAM Germany certified reference material BRM#12-mixed sandy soil

### Data interpretation and statistical analysis

The ability for heavy metal accumulation in soil and translocation upwards in water spinach and okra was determined using translocation factor (TF), accumulation factor (AF) and tolerance index (TI). The TF, AF and TI were estimated as follows: translocation factor (TF) = concentration of metal found in shoot/Concentration of metal found in root; accumulation factor (AF) = concentration of metal found in root/concentration of metal found in soil; and tolerance index (TI) = dry matter yield in spiked metal soil/dry matter yield in control soil. The experimental data were analyzed by one-way analysis of variance (ANOVA) to evaluate the growth performance and the accumulation of metals in both water spinach and okra. Further statistical validity test for significant difference among treatment means was carried out using Fisher’s least significant difference (LSD) with considering the level of significance at (*p* = 0.01).

## Results

### Soil characterization

**Fig. 1:**
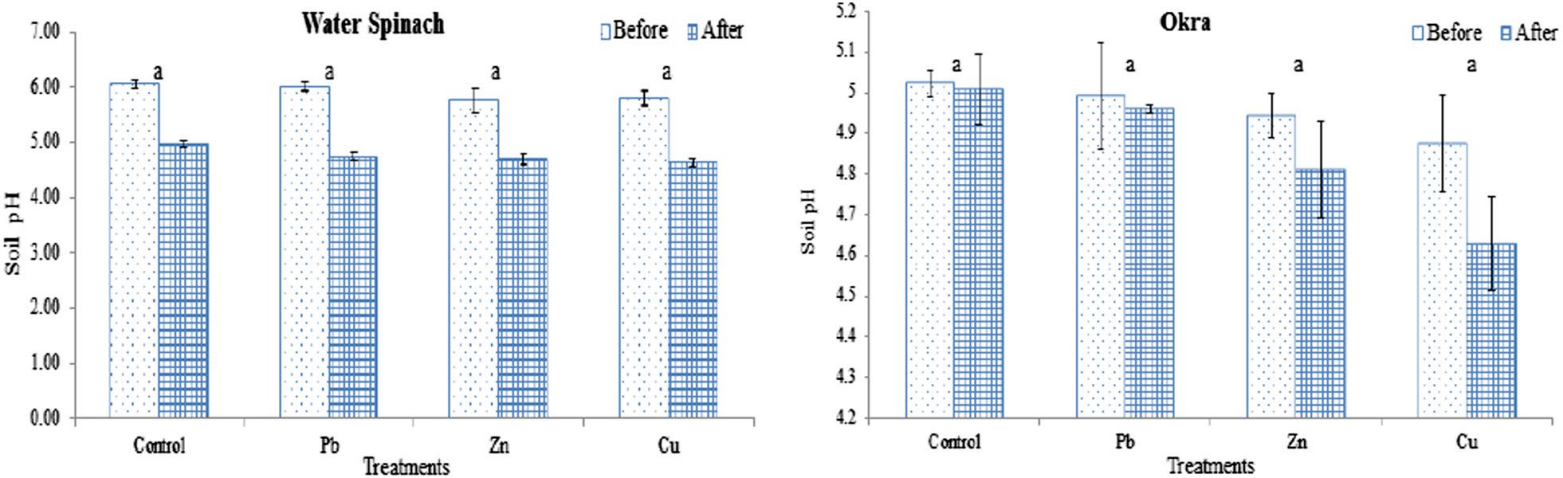
Changes in soil pH in water spinach and okra as influenced by different treatments. *Vertical bars* represent ± standard deviation in treatment means and *same letters* are not significantly different at 0.01 levels of probability

Before planting, the pH of the soil varied from 4.88 to 6.08, where the control soil in water spinach recorded the highest pH of 6.08 while the lowest pH of 4.88 was observed in the okra spiked Cu soil. Upon harvesting, all of the water spinach and okra treatment soils showed a decline in pH ranging from 4.63 to 5.01 where the highest pH reduction (−1.27 pH units) was recorded in spiked Pb of water spinach treatment. However, as can be seen in Fig. 1, there were no significant differences observed between soil pH for water spinach and okra despite the fact that both plants were grown in non-optimum soil pH levels.

### Plant growth performance

**Fig. 2:**
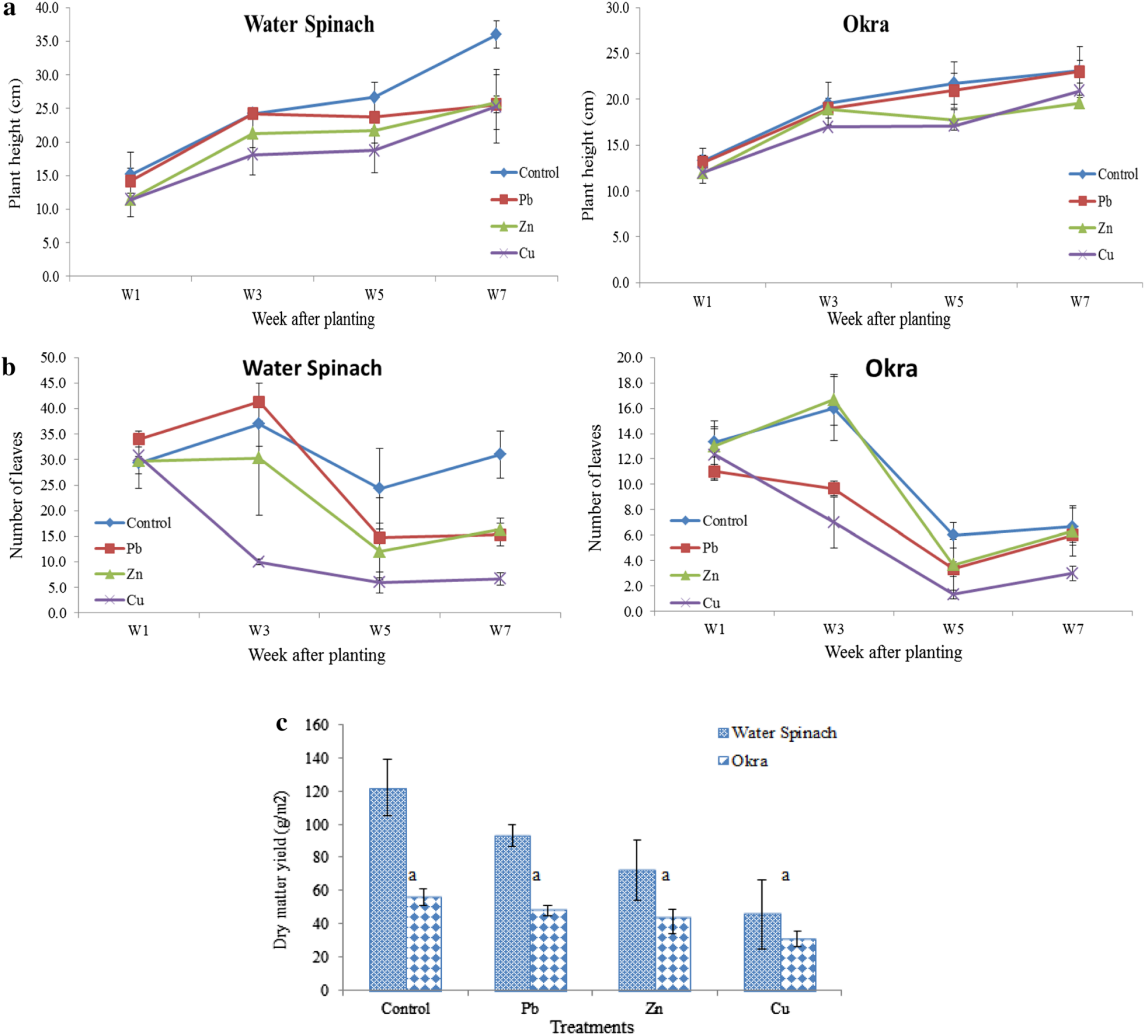
**a** Plant height, **b** number of leaves and **c** Dry matter yield of water spinach and okra as influenced by different types of treatments. *Vertical bars* represent ±standard deviation in treatment means and *same letters* are not significantly different at 0.01 levels of probability

Plant growth performance was monitored by three parameters including plant height, number of leaves and dry matter yield for each treatment. There were no significant differences found between plant height for both water spinach and okra even though all the plants were grown in different spiked metal soils. Both water spinach and okra recorded average plant height ranging from 11.4 to 36.0 cm in all the treatments throughout the growth period (Fig. 2a). However, the picture was different with okra, where the opposite trend was observed, whereby all the okra plants recorded a slower growth compared to water spinach. Furthermore, only the spiked Cu treatment in water spinach showed a significant decreased (*p* < 0.01) in terms of the total number of leaves (Fig. 2b). Okra in spiked Cu treatment recorded the lowest number of leaves with a total average of 1.3 leaves on week 5 while the highest number of 41.3 leaves was observed in water spinach control treatment on week 3. The average number of leaves in water spinach ranged from 6.0 to 41.3, while in okra it ranged between 1.3 and 16.7, throughout the entire growth period. It can be clearly seen that both water spinach and okra grown in the spiked Cu treatment, recorded the lowest number of leaves as compared to other spiked metal treatments. Similarly, with regard to dry matter yield, there were no significant differences found between the dry matter contents of water spinach and okra despite the fact both plants were grown in different spiked metal treatments. The water spinach control treatment recorded the maximum dry matter content per pot (122.08 ± 17.1 g/m^2^) followed by the spiked Pb treatment (93.25 ± 6.7 g/m^2^), while okra grown in the spiked Cu treatment recorded the lowest dry matter content per pot (31.08 ± 4.5 g/m^2^).

### Heavy metal accumulation in plants

Table 3 shows the concentration of metal ions accumulated in the roots and shoots of both water spinach and okra in the different types of spiked metal treatments. Edible sections of shoots in both vegetables include the stems and leaves for water spinach whereas okra contain steams, leaves and fruits. All three Pb, Zn, Cu metals accumulated in different amounts in both water spinach and okra plants. Heavy metal accumulation of Pb, Zn and Cu was significantly higher (*p* < 0.01) in all spiked metal treatments as compared to the control of both water spinach and okra. Between shoot and root; Pb, Zn and Cu accumulation was relatively higher in the roots of both water spinach and okra. Among treatments, Pb treated okra (80.20 ± 4.7 mg/kg) and water spinach (27.69 ± 3.5 mg/kg) recorded a significant increase (*p* < 0.01) of Pb accumulation in the roots as compared to control. Similarly, the Pb accumulation of shoots in Pb treated water spinach (30.31 ± 4.1 mg/kg) and okra (18.51 ± 5.2 mg/kg) also showed significantly higher (*p* < 0.01) with the non-metal spiked control treatment. On the other hand, the highest Zn metal accumulation in water spinach and okra were recorded in the Zn treated plants for both roots and shoots with a significant increase (*p* < 0.01) of Zn metal accumulation to control treatment. All spiked metal treated okra showed significantly higher (*p* < 0.01) when compared to the control treatment for Zn accumulation in the roots. However, only Zn treated water spinach (35.10 ± 2.7 mg/kg) and okra (5.18 ± 1.2 mg/kg) recorded significant increased (*p* < 0.01) with the control treatment for Zn accumulation in the shoots. A significant increase (*p* < 0.01) of Cu metal accumulation was observed among the roots of Cu treated water spinach (34.80 ± 3.4 mg/kg) and okra (10.08 ± 2.4 mg/kg) with the control treatment. The shoots of Cu treated water spinach (18.87 ± 2.6 mg/kg) and okra (2.62 ± 2.4 mg/kg) also showed significantly higher (*p* < 0.01) of Cu metal accumulation with the control treatment. And between shoot and root, a greater accumulation of Cu was observed in the roots of all treatments for both water spinach and okra but the opposite was found in some of the treatments with Pb and Zn metal accumulation. Amongst all three different types of heavy metal, water spinach recorded the highest metal accumulation for Pb (30.31 ± 4.1 mg/kg), Zn (35.10 ± 2.7 mg/kg) and Cu (18.87 ± 2.6 mg/kg) in the shoots of different spiked metal treatments, respectively.

**Table 3:** Accumulation of Pb, Zn and Cu metals (dry weight of mg/kg) in water spinach and okra as influenced by different types of spiked heavy metal treatments

Plant species	Treatment	Pb (mg/kg)			Zn (mg/kg)			Cu (mg/kg)		
		Root	Shoot	Total	Root	Shoot	Total	Root	Shoot	Total
Water spinach	Control	5.67 ± 4.6 b	4.31 ± 4.1 b	9.98 b	1.71 ± 1.6 b	1.42 ± 2.6 b	3.13 b	0.70 ± 1.2 d	0.24 ± 0.4 d	0.94 d
	Pb	27.69 ± 3.5 a	30.31 ± 4.1 a	58.00 a	1.43 ± 0.7 bc	1.26 ± 0.6 bc	2.69 bc	1.70 ± 0.3 c	1.10 ± 0.5 c	2.8 c
	Zn	3.77 ± 1.7 c	3.29 ± 2.3 c	7.06 d	35.70 ± 3.7 a	35.10 ± 2.7 a	70.8 a	4.20 ± 2.0 b	1.52 ± 0.3 b	5.72 b
	Cu	3.92 ± 0.7 c	3.94 ± 1.7 bc	7.86 c	0.94 ± 0.7 bc	1.34 ± 1.7 bc	2.28 c	34.80 ± 3.4 a	18.87 ± 2.6 a	53.6 a
Okra	Control	5.00 ± 1.2 d	4.18 ± 2.9 c	9.18 c	1.10 ± 0.6 d	1.89 ± 1.2 bc	2.99 d	1.03 ± 0.6 c	0.25 ± 0.1 c	1.28 d
	Pb	80.20 ± 4.7 a	18.51 ± 5.2 a	98.71 a	1.31 ± 0.7 b	1.98 ± 2.9 b	3.29 b	1.25 ± 0.7 b	0.43 ± 0.2 b	1.68 b
	Zn	6.12 ± 3.5 b	13.97 ± 2.1 b	20.09 b	9.32 ± 2.9 a	5.18 ± 1.2 a	14.5 a	1.16 ± 0.6 bc	0.23 ± 0.1 c	1.39 c
	Cu	5.25 ± 2.3 bc	4.01 ± 2.9 d	9.26 c	1.23 ± 0.7 c	1.95 ± 1.7 bc	3.18 bc	10.08 ± 2.4 a	2.62 ± 0.7 a	12.7 a

Mean ± standard deviation followed by the same letters is not significantly different for each treatment means at 0.01 levels of probability

### Heavy metal translocation

The different heavy metal accumulation in the roots and shoots of both water spinach and okra are presented together with the associated translocation factor (TF) and accumulation factor (AF) as shown in Table 4. Despite the poor bioavailability of metals in the soil, plants have a high ability to accumulate metals into different plant parts and this may subsequently pose risks to human health especially when the plants are cultivated on or near metal contaminated areas. The amount of metal translocated into different parts of the plant, especially into the edible portion, are crucial and hence, the soil–plant transfer coefficients of the translocation factor (TF) and accumulation factor (AF) have been used to determine the overall metal concentrations in the different plant parts, namely the roots and shoots. The accumulations of Pb and Zn in both water spinach and okra have recorded high TF values. The Zn treated okra recorded TF value of 2.28 in Pb accumulation while control and Cu treated okra recorded 1.72 and 1.59 TF values, respectively in the Zn accumulation. The lowest AF value was observed in Zn treated okra (0.19) of Zn accumulation and the highest AF value was found in Zn treated water spinach (7.65) of Cu accumulation. High TF values of >1 in both water spinach and okra, suggesting that the metal translocation of Pb and Zn from root to shoot was substantial. Furthermore, both water spinach and okra cultivated on all non-metal spiked treatments showed high AF values whilst spiked metal treatments exhibited lower AF values (<1) in the Pb, Zn and Cu treatments.

**Table 4:** Translocation factor (TF), accumulation factor (AF) and tolerance index (TI) of lead, zinc and copper metals in water spinach and okra as influenced by different treatments of control, Pb (50 mg Pb/kg soil), Zn (50 mg Zn/kg soil) and Cu (50 mg Cu/kg soil)

Plant species	Treatment	Pb accumulation		Zn accumulation		Cu accumulation		TI
		TF	AF	TF	AF	TF	AF	
Water spinach	Control	0.76 d	4.61 a	0.83 bc	4.17 a	0.34 c	1.28 c	
	Pb	1.09 a	0.55 d	0.88 bc	3.49 b	0.65 a	3.10 b	0.764 a
	Zn	0.87 c	3.06 c	0.98 b	0.71 d	0.36 c	7.65 a	0.591 ab
	Cu	1.01 b	3.18 b	1.43 a	2.29 c	0.54 b	0.70 d	0.376 ab
Okra	Control	0.84 b	4.06 b	1.72 a	2.68 c	0.24 bc	1.88 c	
	Pb	0.23 d	1.61 d	1.51 bc	3.20 a	0.35 a	2.28 a	0.860 a
	Zn	2.28 a	4.97 a	0.56 d	0.19 d	0.20 d	2.11 ab	0.786 ab
	Cu	0.76 c	4.26 c	1.59 b	3.00 b	0.26 b	0.20 d	0.555 ab

Mean ± standard deviation followed by the same letters is not significantly different for each treatment means at 0.01 levels of probability

### Food and metal contamination

Table 5 shows the permissible levels of soil and food concentration limits for Pb, Zn and Cu. The initial soil concentration of Pb (1.23 mg/kg), Zn (0.41 mg/kg) and Cu (0.55 mg/kg) are below the limit set by the Department of Environment, Malaysia (2009) before all of the metal treatments were spiked with 50 mg/kg of metals and to be used as a contaminated soil. Regardless of the high level of metal spiking in the soil, only Pb accumulation in the shoots of water spinach (30.31 mg/kg) and okra (18.51 mg/kg) exceeded the permissible levels for both the National Malaysia Food Act 1983 and Food Regulations 1985 (2006) and the International Codex Alimentarius Commission–Joint FAO/WHO Expert Committee on Food Additives (JECFA) standards. The international Codex Alimentarius Commission has set the permissible levels of Pb (5 mg/kg), Zn (60 mg/kg) and Cu (40 mg/kg) concentrations in food which are slightly less stringent as compared to the Malaysia Food Act 1983 and Food Regulations 1985 (2006) with the maximum allowable limits of 2, 40 and 30 (mg/kg); respectively for Pb, Zn and Cu metals.

**Table 5:** Permissible levels of Pb, Zn and Cu metals in soil and food standards

Heavy metals	Final soil (mg/kg)		Spiked heavy metal accumulation (mg/kg)		Soil limit (mg/kg)^a^	Food limit (mg/kg)	
	Water spinach	Okra	Water spinach	Okra		Msia^b^	FAO/WHO^c^
Pb	0.622	1.163	30.31	18.51	10.4	2	5
Zn	0.187	0.151	35.10	5.18	21.9	40	60
Cu	0.408	0.094	18.87	2.62	13.8	30	40

^a^Department of Environment (DOE), Malaysia (2009)

^b^Malaysian Food Act 1983 and Food Regulations 1985 (2006)

^c^Codex Alimentarius Commission (1984)

## Discussion

Clay soil normally has a high water holding capacity and nutrient retention, but with low aeration and water infiltration, due to the small particle size of clay soils (Page 1952). The soil pH is also significant being part of the important external environmental parameters that can affect plant growth as well as control the solubility and availability of plant nutrients in the soil. All treatments showed a decrease in soil pH can be likely due to the availability of metal (cation) ions exchange in the plants (Motior et al. 2011, 2013). Generally, most plants are able to grow normally within a certain range of soil, although the rate of plant survival will decline when plants are cultivated in extreme acidic and alkaline conditions. As can be seen from the experimental results, the pH range for both water spinach and okra growth, are in the slightly acidic pH range. Although both plants were able to grow, Moyin-Jesu (2007) reported that a higher soil pH would probably be more suitable to increase the growth and yields of vegetables especially okra.

The possible cause for the lower number of leaves in all spiked metal treatments could be the effects of the added spiked metal solutions in the soils, as inhibitory concentrations will retard metabolism in the plant tissues. Past studies by Yang et al. (2002) and Wang et al. (2013) on Cu metal in vegetables showed that the rate of photosynthesis and plant growth inhibited by the excessive amount of Cu in the plant tissues.

The dry matter content in water spinach was expected to be higher compared to okra due to its higher vegetative growth regardless of the different spiked metal treatments. Okra exhibited minimum dry matter content in all the treatments, as its growth rate was slow throughout the entire experimental period. Dry matter content is an important plant ecology trait which is closely associated with plant growth and survival (Shipley and Vu 2002). Grime and Hunt (1975) have revealed that dry matter content indicates the role of variation in potential relative growth rate and the ecological behaviour of the plant. The possible reason for the slower growth rate observed okra compared to water spinach could be due to the effects of the spiked metals in the contaminated clay soil. The continuous use of contaminated soil may eventually cause both water spinach and okra to have minimum dry matter content in all the treatments, including the control treatment, due to water stagnation problems on clay type soil texture. Soil properties and content are probably the contributing factors which inhibited growth as can be seen in the lower plant height and leaf number in both water spinach and okra regardless of the different types of spiked metal treatments. Thus, the presence of additional spiked metals in the Pb, Zn and Cu treatments contributed to the lower plant growth, specifically in terms of plant height and number of leaves, hence causing a lower output of plant dry matter yield.

Heavy metal accumulation trends for okra were in the order of Pb > Zn > Cu whilst Zn accumulated the highest followed by Pb and Cu in water spinach. The similar trends in Gothberg et al. (2002, 2004) and Huang et al. (2014) experiments recorded appreciably higher accumulation of Pb in water spinach for both treated and untreated Pb treatments. However, variation cultivars of water spinach are likely to have a different range of heavy metal accumulations as recorded by He et al. (2014) and Alia et al. (2015). The high accumulations of Zn and Cu that were found in the roots of both water spinach and okra was possibly due to the translocation these metal ions from soil into the roots because Zn and Cu are required micronutrients that are routinely taken by plants for life processes (Hopkins 1999; Mengel et al. 2001).

TF and AF values are essential indicators that commonly used to evaluate potential plant species for phytoremediation. Nazir et al. (2011) and Malik et al. (2010) reported that both water spinach and okra can be a potential Pb and Zn phyto-accumulators when TF and AF values >1. Due to the high TF and AF values obtained in this study, both water spinach and okra shoots (TF = 1.01–2.28) can be considered to be the sinks (metal accumulators) of Pb and Zn whilst the roots of the water spinach and okra (AF = 1.28–7.65) acting as the sink of accumulation for all three different types of Pb, Zn and Cu metals from the sources of heavy metals in the soil. Both Pb treated water spinach (0.764) and okra (0.860) showed good tolerance properties for Pb metal accumulation which recorded the highest tolerance index (TI) as compared to the plants cultivated under Zn and Cu treatments. Although both Pb treated water spinach and okra recorded high TI, the TF and AF values of water spinach which reflects greater translocation of heavy metals from soil to the shoots of the plant. This scenario could be probably due to the high Pb mobility and good phyto-accumulator properties of water spinach.

Even though, both water spinach and okra cultivated in Zn and Cu treatments did not exceed the allowable levels; the presence of high amounts of Zn and Cu in food is enough to pose health problems, as it has been reported that the major source of these metals are available in almost all urban environmental soils (Thornton 1991; Li et al. 2001). As a general rule of thumb, any high concentration of heavy metal accumulation in the edible parts (shoots of water spinach while okra’s fruit) of vegetables renders it as not recommended for food consumption. Hence, water spinach and okra are not encouraged to grow in soils which are contaminated with Pb as the study has shown that both possessed high Pb accumulating ability aligned with the recommendations as proposed by Gothberg et al. (2002) and Marcussen et al. (2008). Although the concentrations of Zn and Cu for both water spinach and okra were lower than the national and international permitted food standards, continuous monitoring and further research in assessing the accumulation of metals in other different types of vegetables is essential in order to avoid excessive bioaccumulation of hazardous metals as well as ensuring the quality of the vegetables. Nevertheless, the study also recognizes that the use of pot assays instead of field-site experiments would probably give rise to an enhanced accumulation of heavy metals in plants. In addition, the accumulation of heavy metals in plants is extremely complex in nature as various biotic and abiotic factors may likely to influence the mechanisms of phytoremediation. It would be worthwhile extending this study to other metal elements and a wider range of commonly grown vegetables.

## Conclusions

The accumulation of Pb, Zn and Cu varied between both plants. For okra, heavy metal accumulations were in the order of Pb > Zn > Cu whilst Zn accumulated the highest followed by Pb and Cu in water spinach. It highlights that there were significant differences (*p* < 0.01) found in both water spinach and okra cultivated under the three spiked metal treatments of Pb, Zn and Cu. It also indicated that both water spinach and okra shoots are the sinks for Pb and Zn accumulation while the roots acted as the sink for the heavy metal accumulation of all three metal ions, Pb, Zn and Cu metals due to its high TF and AF values. Among all the three different spiked metal treatments, both water spinach and okra showed great tolerance for Pb accumulation as the accumulation of Pb was high in the roots and shoots of both plants. The concentrations of Pb in the shoots of water spinach and okra exceeded maximum permissible levels of the national Malaysian Food Act 1983 and Food Regulations 1985 (2006) as well as the international Codex Alimentarius Commission limits. The variation of Pb metal accumulation between the different parts of plants may be useful for selecting suitable cultivation of vegetable species in order to minimize its intake of potentially harmful elements. Hence, the study strongly suggests that water spinach and okra are not recommended to be cultivated in Pb contaminated soils.
